# *YES1* amplification confers trastuzumab–emtansine (T-DM1) resistance in HER2-positive cancer

**DOI:** 10.1038/s41416-020-0952-1

**Published:** 2020-06-23

**Authors:** Lei Wang, Quanren Wang, Piaopiao Xu, Li Fu, Yun Li, Haoyu Fu, Haitian Quan, Liguang Lou

**Affiliations:** Shanghai Institute of Materia Medica, Chinese Academy of Sciences, 201203 Shanghai, China

**Keywords:** Cancer therapeutic resistance, Breast cancer

## Abstract

**Background:**

Trastuzumab–emtansine (T-DM1), one of the most potent HER2-targeted drugs, shows impressive efficacy in patients with HER2-positive breast cancers. However, resistance inevitably occurs and becomes a critical clinical problem.

**Methods:**

We modelled the development of acquired resistance by exposing HER2-positive cells to escalating concentrations of T-DM1. Signalling pathways activation was detected by western blotting, gene expression was analysed by qRT-PCR and gene copy numbers were determined by qPCR. The role of Yes on resistance was confirmed by siRNA-mediated knockdown and stable transfection-mediated overexpression. The in vivo effects were tested in xenograft model.

**Results:**

We found that Yes is overexpressed in T-DM1–resistant cells owing to amplification of chromosome region 18p11.32, where the *YES1* gene resides. Yes activated multiple proliferation-related signalling pathways, including EGFR, PI3K and MAPK, and led to cross-resistance to all types of HER2-targeted drugs, including antibody-drug conjugate, antibody and small molecule inhibitor. The outcome of this cross-resistance may be a clinically incurable condition. Importantly, we found that inhibiting Yes with dasatinib sensitised resistant cells in vitro and in vivo.

**Conclusions:**

Our study revealed that *YES1* amplification conferred resistance to HER2-targeted drugs and suggested the potential application of the strategy of combining HER2 and Yes inhibition in the clinic.

## Background

HER2 is amplified in up to 20% of primary breast cancers and is associated with poor patient prognosis.^1^ Monoclonal antibodies that target HER2, such as trastuzumab and pertuzumab, as well as small molecule kinase inhibitors, such as lapatinib and neratinib, which target both HER2 and EGFR, have been approved by the Food and Drug Administration (FDA) for the treatment of HER2-positive cancer.^2^ However, despite initial responses to these agents, acquired resistance almost universally develops.^3^

The development of trastuzumab–emtansine (T-DM1), an antibody-drug conjugate (ADC) comprising trastuzumab and the antimicrotubule maytansinoid drug emtansine (DM1), brought new hope for HER2-positive patients. T-DM1 possesses the biological properties of trastuzumab, namely HER2-signaling inhibition and antibody-dependent cell-mediated cytotoxicity, as well as the antimicrotubule activity of DM1.^4^ T-DM1 has been reported to overcome resistance to trastuzumab^5^ and lapatinib^6^ in HER2-amplified cancer cells, and was approved for HER2-positive metastatic breast cancer by the FDA based on the phase III EMILIA trial (NCT00829166).^7,8^

However, although T-DM1 produces enormous clinical benefits, some patients do not respond to the drug or ultimately develop acquired resistance.^9^ Identified mechanisms of T-DM1 resistance include defects in T-DM1 binding,^10^ trafficking^11,12^ and microtubule dynamics.^13,14^ These resistance factors play a critical role in the effects of T-DM1, but not those of non-ADC anti-HER2 drugs, and thus may be overcome by other HER2-targeted drugs. Indeed, we have reported that lapatinib overcomes T-DM1 resistance in a cell line with impaired lysosomal proteolytic degradation of T-DM1.^11^ However, beyond that, cross-resistance mechanisms of T-DM1 with other HER2-targeted drugs, which may lead to an incurable clinical condition, are poorly characterised. Therefore, a better understanding of T-DM1-resistance mechanisms, especially of cross-resistance mechanisms, is particularly important.

In this study, which is part of our continuing effort to address the problem of resistance to HER2-targeted drugs,^11,15,16^ we found that the non-receptor tyrosine kinase Yes is overexpressed in a cell line with acquired resistance to T-DM1 owing to amplification of chromosome region 18p11.32, where *YES1* gene resides. Notably, this overexpression of Yes conferred cross-resistance to all types of HER2-targeted drugs. We further suggest the possible therapeutic strategy of combining HER2 with Yes inhibition for overcoming resistance to HER2-targeted drugs.

## Methods

### Reagents and antibodies

T-DM1 and trastuzumab were purchased from F. Hoffmann-La Roche (Basel, Switzerland). Gefitinib, AZD4547, crizotinib, sunitinib, imatinib, dasatinib, PD 0325901 and GDC-0941 were purchased from Selleck Chemicals (Houston, TX, USA). DM1 was purchased from Meilunbio Inc. (Dalian, China). T-DM1 and trastuzumab were dissolved in saline, and small molecule compounds were dissolved in dimethyl sulfoxide. Lyso-Tracker Deep Red and DyLight 488 NHS ester were purchased from Thermo-Fisher Scientific (Waltham, MA, USA). Sulforhodamine B and the antibody against β-tubulin were purchased from Sigma–Aldrich (St. Louis, MO, USA). Antibodies against phospho-HER2 (Tyr1221/1222), HER2, phospho-EGFR (Tyr845), EGFR, phospho-HER3 (Tyr1289), HER3, phospho-Met (Tyr1234/1235), phospho-IGF-IR (Tyr1135/1136)/IR (Tyr1150/1151), phospho-Akt (Ser473), phospho-Erk1/2 (Thr202/Tyr204), phospho-PTEN (Ser380/Thr382/383), PTEN, phospho-Src family kinase (Tyr416), c-Src, Yes, Fyn, Lyn, Lck, Csk and PARP were purchased from Cell Signaling Technology (Beverly, MA, USA). Antibodies against Erk1/2, p27, TYMS, and THOC1 were purchased from Santa Cruz Biotechnology (Santa Cruz, CA, USA).

### Cell culture and treatment

The human BT-474 and SK-OV-3 cell lines were obtained from the American Type Culture Collection (Manassas, VA, USA) and were cultured according to the instructions provided. Acquired T-DM1-resistant cells (BT-474/R1-7) were established by exposing parental BT-474 cells to increasing concentrations of T-DM1 (from 10 ng/mL to 1 μg/mL) for 12 months and selecting clones using the limiting dilution method. BT-474 and SK-OV-3 cells expressing *YES1 Y537F* and *YES1 WT* were generated by transfecting cells with the YES1 Y537F (Addgene plasmid #51299)^17^ and YES1 WT (generated by point mutation from *YES1 Y537F* plasmid) plasmids, respectively.

### Lentiviral overexpression

YES1 Y537F or YES1 WT together with psPAX2 and pMD2G plasmids were transfected into HEK293FT cells in a 4:3:1 ratio using Lipofectamine 2000 (Thermo-Fisher Scientific, Waltham, MA, USA). Lentiviral supernatants were harvested after 48 and 72 h. BT-474 and SK-OV-3 cells were transfected with lentiviral supernatants at 37 °C. 24 h later, the viral supernatants were removed and cells were cultured in the presence of blasticidin (BT-474: 20 μg/ml; SK-OV-3: 10 μg/ml) for 7 days.

### Cell proliferation assay

Cells were treated with different concentrations of drugs, alone or in combination, as indicated, inhibition rates were determined using sulforhodamine B assays and 50% growth-inhibition concentration (IC_50_) was calculated using GraphPad Prism software, as described previously.^18^

### Western blotting

Western blotting was performed using standard procedures, as described previously.^19^ Briefly after drug treatment, cells were harvested and cell lysates were separated by SDS–PAGE and transferred to polyvinylidene difluoride membranes. After blocking in 5% nonfat milk in TBST (Tris-buffered saline containing 0.1% Tween-20, pH 7.6), membranes were incubated with primary and secondary antibodies. Immunoreactive proteins were visualised using the enhanced chemiluminescence system from Thermo-Fisher Scientific (Waltham, MA, USA). Results were quantified by densitometry and normalised to corresponding total protein (for phosphorylated protein) or β-tubulin control.

### Binding and endocytosis assay

Binding and endocytosis assays were performed as described previously.^15^ For binding assay, cells were incubated with DyLight 488 NHS-ester-linked T-DM1 on ice for 1 h. For endocytosis assay, cells were incubated with DyLight 488 NHS-ester-linked T-DM1 at 37 °C for different indicated time and subsequently stripped using a stripping buffer (0.05 mol/L glycine, 0.1 mol/L NaCl, pH 2.45). Fluorescence was finally analysed by flow cytometry.

### Fluorescence microscopy

Cells were incubated with DyLight 488 NHS-ester-linked T-DM1 for 23 h at 37 °C and the lysosome fluorescent probe Lyso-Tracker Red was added for another 1 h. Alternatively, cells were incubated with Lyso-Tracker Red for 1 h at 37 °C, then incubated with DyLight 488 NHS-ester-linked T-DM1 for 10 min at 4 °C. Cells were fixed with 4% paraformaldehyde for 15 min and imaged with an Olympus FV1000 confocal microscope.

### Polymeric tubulin fraction assay

Cells was lysed with a buffer consisting of 80 mmol/L MES-KOH (pH 6.8), 1 mmol/L MgC1_2_, 1 mmol/L EGTA, 0.1% Triton X-100 and 10% glycerol for 3 min at 30 °C, and detergent-insoluble polymerised cytoskeleton was analysed by western blotting.

### In vivo study

BT-474/R1-7 tumour models were established by subcutaneous inoculation of BT-474/R1-7 cells into female Balb/cA-nude mice (5–6 weeks old, purchased from Shanghai SLAC Laboratory Animal Co. Ltd), and SK-OV-3/YES1 Y537F tumour models were established by subcutaneous inoculation of SK-OV-3/YES1 Y537F cells into female NCG mice (5–6 weeks old, purchased from Jiangsu GemPharmatech Co. Ltd). Animals were bred in specific-pathogen-free environment under standard conditions (temperature: 22 ± 1 °C; dark/light cycle: 12/12 h; humidity: 60 ± 10%; cage: plastic, sterile, with filter; cage companions: max 6 animals/cage; bedding material: high-adsorbing bedding material without dust). Tumour diameter was measured by vernier calliper. When tumours reached a volume of 100–300 mm^3^, mice were randomised into control (*n* = 10) or treatment (*n* = 6) groups and treated with drugs. Control group: saline, 10 mL/kg, intravenous (i.v.), day 0, 7, 14, 29 (BT-474/R1-7) or day 0, 7, 20 (SK-OV-3/YES1 Y537F). T-DM1 group: 3 mg/kg, i.v., day 0, 7, 14, 29 (BT-474/R1-7) or day 0, 7, 20 (SK-OV-3/YES1 Y537F). Dasatinib group: 3 mg/kg, intragastric (i.g.), once a day. Combination group: T-DM1 (described above) plus dasatinib (described above). T-DM1 was diluted in saline and dasatinib was diluted in citric acid buffer (0.1% Tween 80, pH4.6). Tumour volume was calculated as (length × width^2^)/2, and body weight was monitored as an indicator of general health twice a week. Mice were euthanised by cervical dislocation at the end of the experiments (24 h after the last dose of T-DM1 and 4 h after the last dose of dasatinib), and the tumours were resected and frozen in liquid nitrogen. Tumour growth inhibition (%) was calculated as 100 − (T_t_ − T_0_)/(C_t_ − C_0_) × 100, where T_t_ is the mean volume of treated tumour at time t, T_0_ is the mean tumour volume immediately prior to treatment (time 0), C_t_ is the mean tumour volume of controls at time t, and C_0_ is the mean tumour volume in controls at time 0. Animal experiments were carried out in accordance with guidelines of the Institutional Animal Care and Use Committee at the Shanghai Institute of Materia Medica, Chinese Academy of Sciences.

### Statistical analysis

Data were analysed using GraphPad Prism software. Two-tailed Student’s *t*-tests were used to determine the statistical significance of differences between two groups. A *p*-value < 0.05 was considered statistically significant.

### Additional methods

Methods for siRNA transfection, RNA isolation, quantitative reverse transcription-PCR (qRT-PCR) and qPCR are provided in Supplementary Material.

## Results

### BT-474/R1-7 cells are resistant to HER2-targeted drugs

**Fig. 1:**
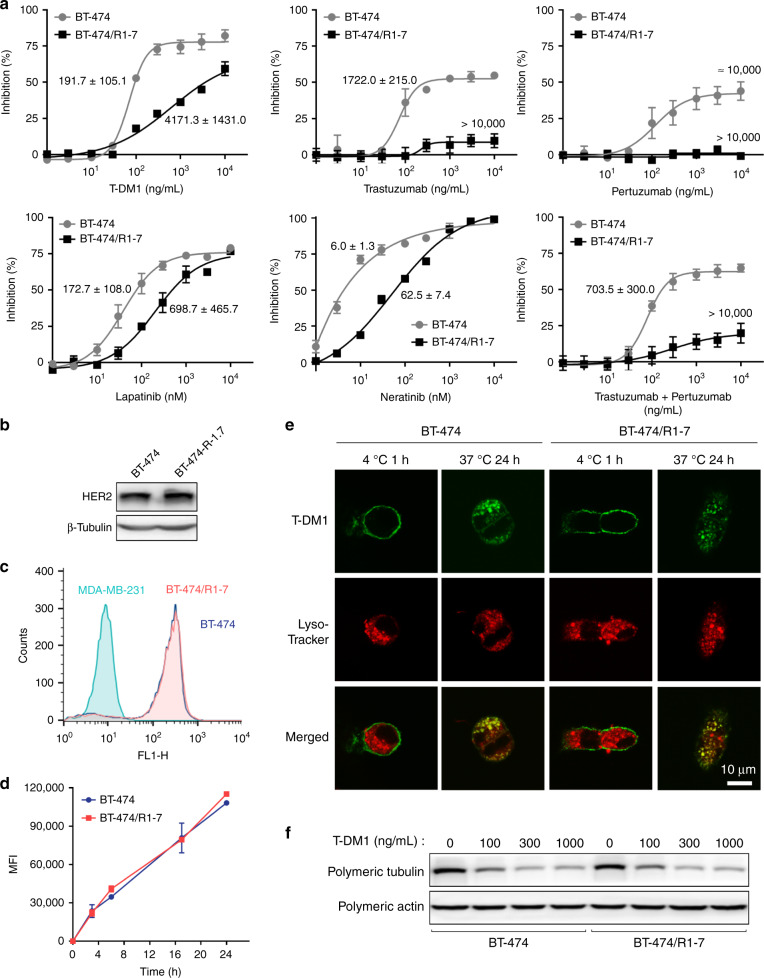
BT-474/R1-7 cells are resistant to HER2-targeted drugs. **a** Cells were treated with different concentrations of T-DM1, trastuzumab, pertuzumab, lapatinib, neratinib or the combination of trastuzumab and pertuzumab for 120 h, after which cell survival was measured using sulforhodamine B assays. IC_50_ was exhibited close to the corresponding dose-response curve. Data shown represent means ± SD of three independent experiments. **b** HER2 levels were determined by western blotting. **c**, **d** Cells were incubated with DyLight 488 NHS-ester–labelled T-DM1 (1 μg/mL) on ice for 1 h (**c**), or at 37 °C for the indicated times, followed by quenching of surface fluorescence with stripping buffer (**d**). Mean fluorescence intensity (MFI) was determined by flow cytometry. **e** Cells were incubated with DyLight 488 NHS-ester–labelled T-DM1 (1 μg/mL, green), and lysosomes were labelled with Lyso-Tracker (100 nM, Red). Samples were visualised by confocal microscopy. Colour figures were shown in html full text version. **f** Cells were treated with T-DM1 for 48 h, after which polymeric tubulin was examined by western blotting.

To investigate the molecular mechanism of T-DM1 resistance, we established T-DM1–resistant clone BT-474/R1-7 from the HER2-positive breast cancer cell line BT-474 following repeated treatment with T-DM1. The degree of T-DM1 resistance in BT-474/R1-7 cells was evaluated using cell-growth assays. As shown in Fig. 1a, the IC_50_ value of T-DM1 in BT-474/R1-7 cells was ~22-fold higher than that in BT-474 cells, indicating significant resistance to T-DM1. We also investigated the sensitivity of BT-474/R1-7 cells to other HER2-targeted drugs, including the antibody drugs trastuzumab and pertuzumab, and the small molecule inhibitors lapatinib and neratinib, and found that BT-474/R1-7 cells were significantly resistant to either drug alone or the combination of trastuzumab and pertuzumab (Fig. 1a). These data suggest that BT-474/R1-7 cells are resistant to different types of HER2-targeted drugs, including antibodies, ADCs and small molecule inhibitors.

### HER2 expression, T-DM1 trafficking and microtubule dynamics are not changed in BT-474/R1-7 cells

Normally, T-DM1 binds to HER2, is endocytosed into cells and releases DM1 through proteolytic degradation, ultimately leading to inhibition of microtubule assembly.^20^ Because factors that affect these processes may play a role in T-DM1 resistance,^20^ we first investigated HER2 expression and T-DM1 drug-release processes in BT-474/R1-7 cells. We found that HER2 levels (Fig. 1b, Supplementary Fig. 1a), as well as T-DM1 binding (Fig. 1c), endocytosis (Fig. 1d) and location (Fig. 1e), were not changed in BT-474/R1-7 cells. We then assessed inhibition of microtubule polymerisation, a critical outcome elicited by DM1-containing catabolites. As shown in Fig. 1f and Supplementary Fig. 1b, T-DM1 caused a concentration-dependent inhibition of tubulin polymerisation in BT-474/R1-7 cells, indicating that microtubule dynamics and the release of DM1-containing catabolites through proteolytic degradation were also not defective in BT-474/R1-7 cells. Taken together, these results suggest that HER2 expression, T-DM1 trafficking and microtubule dynamics are not changed, and are thus not the cause of T-DM1 resistance in BT-474/R1-7 cells.

### Src family kinases play a critical role in drug resistance in BT-474/R1-7 cells

**Fig. 2:**
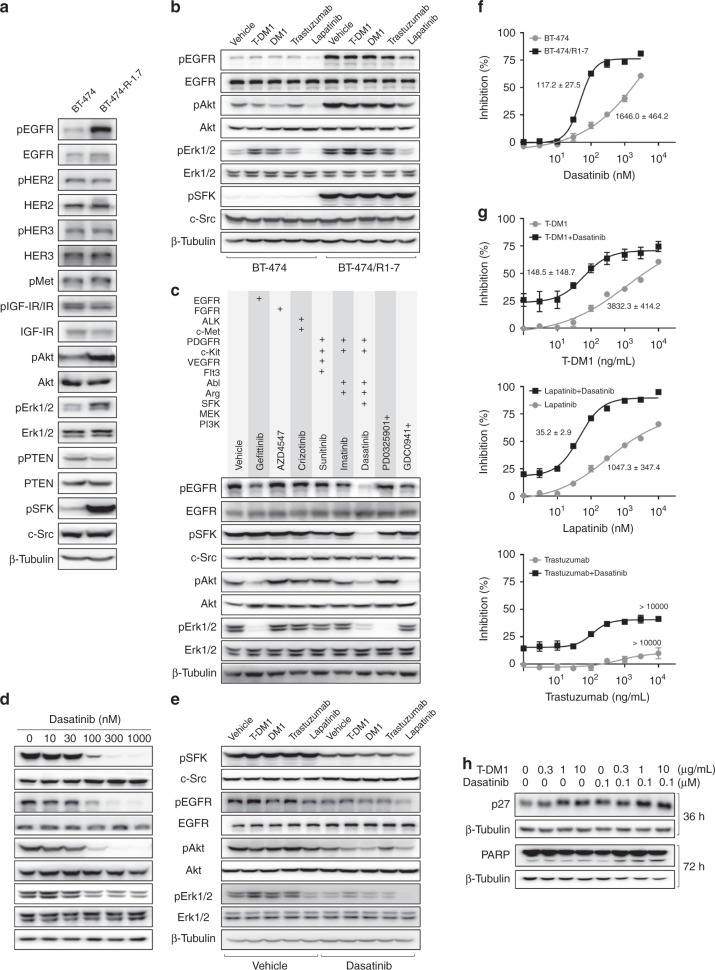
SFK plays a critical role in mediating resistance in BT-474/R1-7 cells. **a** Signalling proteins in BT-474 and BT-474/R1-7 cells were determined by western blotting. **b**–**e** BT-474/R1-7 cells were treated with the indicated inhibitors (T-DM1 and trastuzumab: 1 μg/mL; DM1: 30 nM; lapatinib: 300 nM; other inhibitors: 1 μM or used as indicated) for 2 h, after which the phosphorylation status of signalling proteins was determined by western blotting. **f**, **g** BT-474 and BT-474/R1-7 cells were treated with different concentrations of inhibitors alone or combined with dasatinib (30 nM) for 120 h, after which cell survival was measured using sulforhodamine B assays. Data shown represent means ± SD of three independent experiments. **h** BT-474/R1-7 cells were treated with the indicated inhibitors for 36 or 72 h, after which proteins were determined by western blotting.

HER2-targeted drugs inhibit several proliferation-related signalling pathways in HER2-positive cells, and compensatory activation of such signalling pathways may lead to resistance. Accordingly, we next examined the status of signalling proteins in BT-474/R1-7 cells. As shown in Fig. 2a and Supplementary Fig. 1c, phosphorylation of HER2, HER3, c-Met, IGF-IR, IR and PTEN were not changed, but phosphorylation of EGFR, Akt, Erk1/2 and Src family kinases (SFKs) were significantly increased in BT-474/R1-7 cells. Moreover, the inhibitory effects of HER2-targeted drugs on the phosphorylation of signalling proteins in BT-474 cells were almost lost in BT-474/R1-7 cells (Fig. 2b and Supplementary Fig. 1d), suggesting that reprogramming of these signalling pathways may be a mechanism of acquired resistance. To determine the identity of the aberrantly activated signalling protein that lies upstream in this mechanism, we tested various inhibitors for their ability to suppress activation of all signalling proteins. As shown in Fig. 2c and Supplementary Fig. 1e, dasatinib, which mainly targets SFKs and Abl, and also inhibits Arg, c-Kit and PDGFR,^21^ inhibited all activated signalling proteins (SFK, EGFR, Akt and Erk1/2) in BT-474/R1-7 cells, an effect that was concentration dependent (Fig. 2d and Supplementary Fig. 1f). However, imatinib, an inhibitor of Abl, Arg, c-Kit and PDGFR, but not SFKs,^22^ had no inhibitory effects on these proteins. Besides, gefitinib (EGFR inhibitor) inhibited phosphorylation of EGFR, and its downstream signalling proteins (Akt and Erk1/2), but not phosphorylation of SFKs; GDC-0941 (PI3K inhibitor) and PD 0325901 (MEK inhibitor) only inhibited their respective signalling without affecting other signalling tested. These results suggested that SFK is the most upstream signalling element in the cascade of aberrantly activated signalling proteins in BT-474/R1-7 cells. We then investigated the role of SFK in the unresponsiveness of BT-474/R1-7 cells to the effects of HER2-targeted drugs. As shown in Fig. 2e and Supplementary Fig. 1g, dasatinib decreased aberrant activation of all signalling proteins in this cascade, and restored the inhibitory effects of HER2-targeted drugs. We further explored the role of SFKs in drug resistance using proliferation assays. In contrast to their resistance to HER2-targeted drugs, BT-474/R1-7 cells were significantly more sensitive to dasatinib than BT-474 cells (Fig. 2f). Moreover, a low concentration of dasatinib (30 nM) considerably sensitised BT-474/R1-7 cells to HER2-targeted drugs (Fig. 2g). To further explore the mechanism of cell death, we then tested p27, a marker of cell-cycle arrest, and cleaved PARP, a marker of apoptosis, in BT-474/R1-7 cells. As shown in Fig. 2h and Supplementary Fig. 1h, when combined with dasatinib, T-DM1 induced a more obvious increase of p27 and cleaved PARP, indicating that a stronger cell-cycle arrest and subsequent apoptosis was induced by this combination. Collectively, these results suggest that SFKs, primary targets of dasatinib, play a critical role in drug resistance in BT-474/R1-7 cells.

### Yes, but not c-Src, is responsible for activation of signalling pathways in BT-474/R1-7 cells

**Fig. 3:**
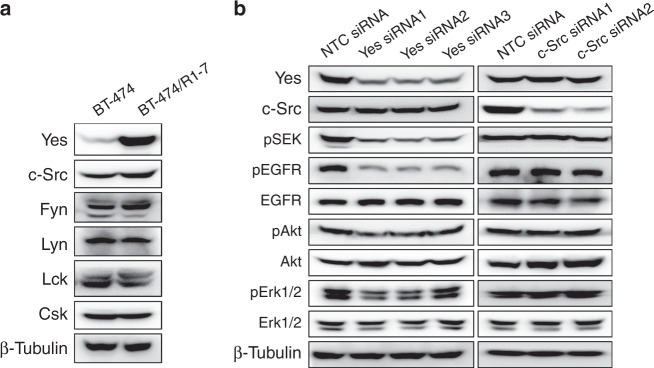
Yes is responsible for aberrant activation of signalling pathways in BT-474/R1-7 cells. **a** Expression of SFK members in BT-474 and BT-474/R1-7 cells was determined by western blotting. **b** BT-474/R1-7 cells were transfected with non-targeting control (NTC) siRNA, Yes siRNAs or c-Src siRNAs for 48 h. Signalling proteins were determined by western blotting.

The SFK family comprises nine members—c-Src, Yes, Fyn, Lyn, Lck, Fgr, Blk, Hck and Yrk—that share similar structure and function.^23^ c-Src, Yes and Fyn are expressed ubiquitously, whereas the other SFKs are more or less tissue specific.^24^ To identify the specific SFK involved in the resistance to T-DM1, we determined the levels of individual SFK members in BT-474/R1-7 cells. As shown in Fig. 3a and Supplementary Fig. 2a, Yes expression was significantly increased in BT-474/R1-7 cells (*P* < 0.001), but the expression of other SFK members (c-Src, Fyn, Lyn and Lck) and Csk, a kinase that negatively regulates SFKs, were largely unchanged. To explore the role of Yes in resistance, we transfected BT-474/R1-7 cells with three independent small interfering RNAs (siRNAs) targeting Yes and assessed aberrant activation of SFK, EGFR, Akt and Erk1/2 signalling pathways. As shown in Fig. 3b and Supplementary Fig. 2b, Yes knockdown selectively inhibited Yes expression (*p* < 0.01) and significantly inhibited all activated signalling proteins (SFK, EGFR, Akt and Erk1/2), suggesting that Yes overexpression is the upstream event in the aberrant activation of these signalling pathways. Because c-Src is the predominant member of SFKs, and has been considered the common node of trastuzumab resistance,^25^ we also knocked down c-Src using siRNAs. As shown in Fig. 3b, c-Src knockdown decreased c-Src expression to a low level, but had no discernible effects on activation of SFK, EGFR, Akt or Erk1/2, suggesting that c-Src is not involved in aberrant signalling pathway activation in BT-474/R1-7 cells. Collectively, these results highlight a critical role for Yes in mediating drug resistance.

### Chromosome region 18p11.32 is amplified in BT-474/R1-7 cells

**Fig. 4:**
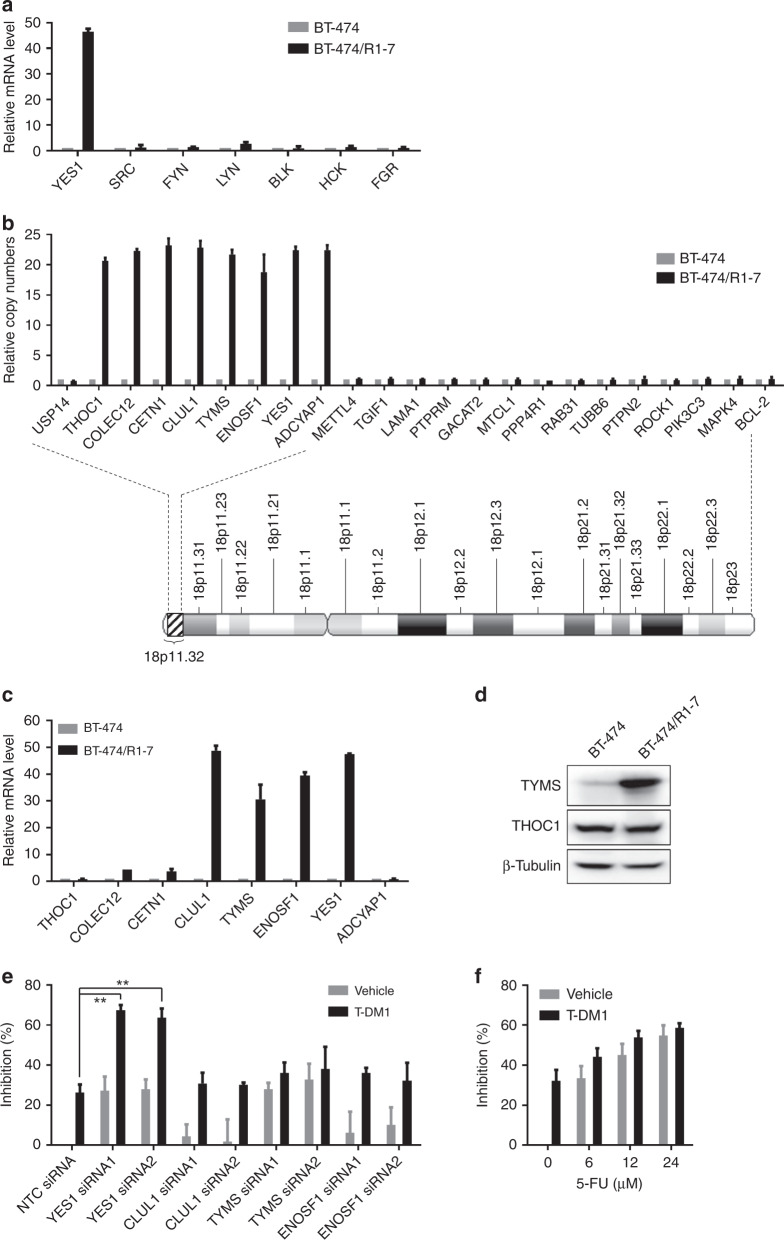
Chromosome region 18p11.32 is amplified in BT-474/R1-7 cells. mRNA levels of SFK members (**a**) and other genes in the 18p11.32 region (**c**) were analysed by qRT-PCR. **b** Copy numbers of genes on chromosome 18 were analysed by qPCR. **d** Protein levels of TYMS and THOC1 were determined by western blotting. **e** BT-474/R1-7 cells were transfected with siRNAs for 24 h, and then treated with T-DM1 for an additional 120 h. **f** BT-474/R1-7 cells were treated with different concentrations of 5-FU, with or without T-DM1 (300 ng/mL), for 120 h. Cell survival in **e**, **f** was measured using sulforhodamine B assays. Data shown represent means ± SD (error bars) from triplicates (**p < 0.01).

To understand the mechanism underlying Yes overexpression, we further examined Yes expression at the mRNA level. As shown in Fig. 4a, *YES1* mRNA was dramatically increased (~46-fold, *p* < 0.0001) in BT-474/R1-7 cells, whereas mRNAs of other SFK members (*SRC*, *FYN*, *LYN*, *BLK*, *HCK* and *FGR*) were largely unchanged. A further examination of DNA copy number showed that the *YES1* gene was significantly amplified (~22-fold, *p* < 0.0001) in BT-474/R1-7 cells (Fig. 4b), suggesting that Yes overexpression is attributable to changes at the genomic level. Because gene amplification usually occurs in the form of an increase in the copy number of a chromosome region,^26^ we further analysed whether other genes located near *YES1* were amplified in BT-474/R1-7 cells. This analysis showed that the copy numbers of seven genes—*THOC1*, *COLEC12*, *CETN1*, *CLUL1*, *TYMS*, *ENOSF1* and *ADCYAP1*—in the 18p11.32 region of chromosome 18 were significantly amplified (Fig. 4b). A further examination of the expression of these genes at mRNA and protein levels showed that *CLUL1*, *TYMS* and *ENOSF1* mRNA levels were also significantly increased (Fig. 4c), and confirmed TYMS overexpression at the protein level (Fig. 4d and Supplementary Fig. 2, c). We then asked whether overexpression of these genes was associated with drug resistance. To this end, we knocked down *CLUL1*, *TYMS*, *ENOSF1* or *YES1* using two independent siRNAs for each, and determined whether the resulting knockdown sensitised BT-474/R1-7 cells to T-DM1 treatment. Because TYMS is the target for the chemotherapeutic agent 5-fluorouracil (5-FU), we also assessed the effects of 5-FU on resistance to T-DM1. These analyses showed that only *YES1* knockdown reversed T-DM1 resistance in BT-474/R1-7 cells (Fig. 4e); siRNA-mediated knockdown of TYMS or inhibition with 5-FU exerted inhibitory effects on cell growth as broad cytotoxic drugs did, but these treatments did not reverse T-DM1 resistance (Fig. 4e, f and Supplementary Fig. 3). Thus, among the amplified genes in the 18p11.32 region, *YES1* is the critical gene for T-DM1 resistance.

### Ectopic expression of Yes confers resistance to HER2-targeted drugs

**Fig. 5:**
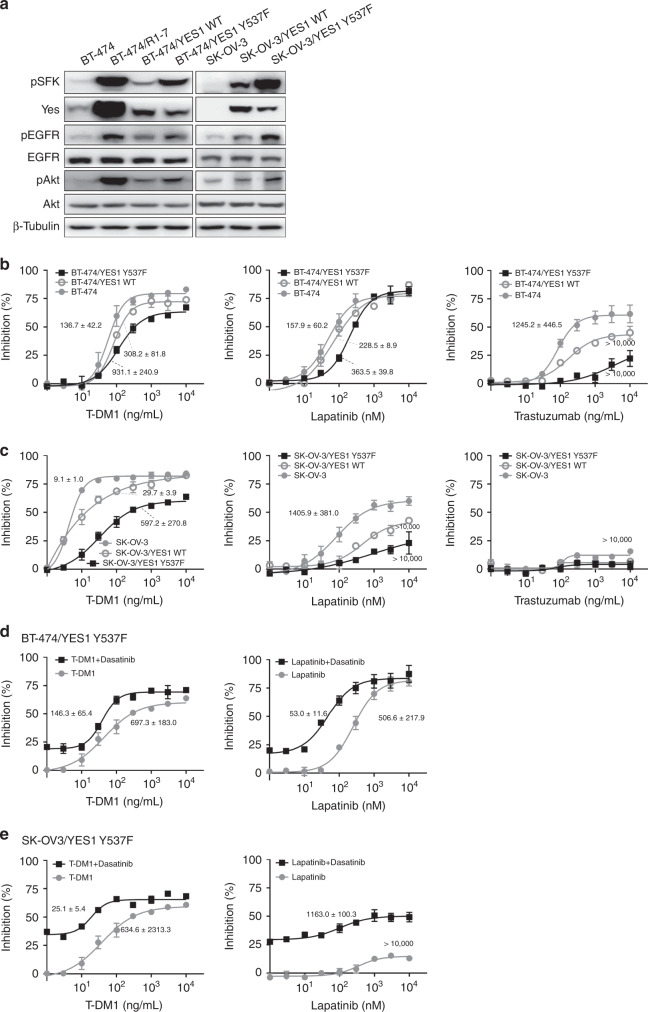
Ectopic expression of Yes confers T-DM1 resistance. **a** Signalling proteins in indicated cells were determined by western blotting. BT-474, BT-474/YES1 WT and BT-474/YES1 Y537F cells (**b**), and SK-OV-3, SK-OV-3/YES1 WT and SK-OV-3/YES1 Y537F cells (**c**), were treated with different concentrations of T-DM1, trastuzumab or lapatinib for 120 h, after which cell survival was measured using sulforhodamine B assays. BT-474/YES1 Y537F (**d**) and SK-OV-3/YES1 Y537F (**e**) cells were treated with different concentrations of the HER2-targeted inhibitors, T-DM1 or lapatinib, for 120 h, with or without dasatinib (30 nM), after which cell survival was measured using sulforhodamine B assays. Data shown represent means ± SD of three independent experiments.

To determine whether Yes activation is sufficient to confer resistance to HER2-targeted drugs, we stably transfected BT-474 breast cancer cells (parental cells of BT-474/R1-7) and SK-OV-3 ovarian cancer cells (to assess cell specificity) with wild-type *YES1* (WT) or constitutively active *YES1* (Y537F), and analysed resistance to the HER2-targeted drugs. Transfection of *YES1 WT* and *YES1 Y537F* led to similar protein levels of Yes in both BT-474 and SK-OV-3 cell models, but *YES1 Y537F* led to a stronger activation of signalling proteins (Fig. 5a and Supplementary Fig. 2d). Although ectopic expression of Yes in BT-474 cells did not achieve equivalent level as that in BT-474/R1-7 cells, activation of signalling proteins in BT-474/YES1 Y537F cells was comparable to that in BT-474/R1-7 cells. *YES1 WT* and *YES1 Y537F* conferred cell resistance to all HER2-targeted drugs tested (T-DM1, trastuzumab and lapatinib), and the resistance caused by *YES1 Y537F* was stronger (Fig. 5b, c). We further examined whether dasatinib was capable of reversing resistance to these HER2-targeted drugs in these cell models as it did in BT-474/R1-7 cells. As shown in Fig. 5d and e, dasatinib treatment sensitised resistant cells to HER2-targeted drugs in both cell models. Collectively, these observations indicate that Yes amplification confers resistance to HER2-targeted drugs, and the resistance level mainly depends on the activation degree of Yes.

### Yes inhibition overcomes T-DM1 resistance in vivo

**Fig. 6:**
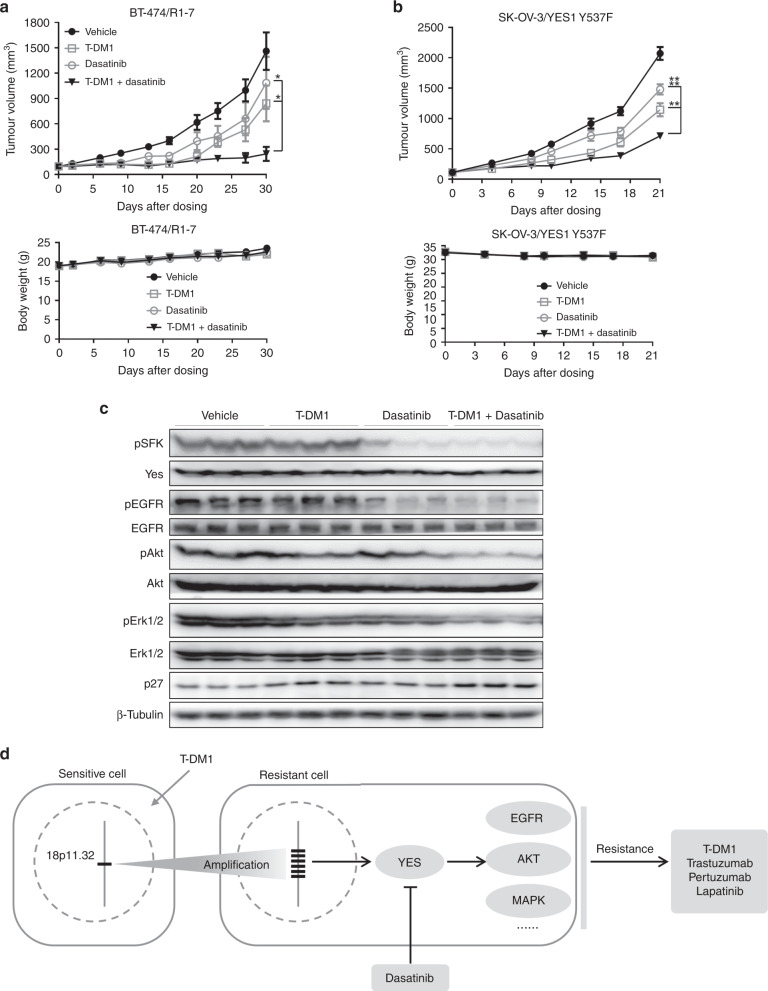
Yes inhibition overcomes T-DM1 resistance in vivo. Mice bearing BT-474/R1-7 (**a**) or SK-OV-3/YES1 Y537F (**b**) xenograft tumours were treated with T-DM1, dasatinib, or a combination of T-DM1 and dasatinib. Tumour volumes (top) and body weights (bottom) were measured on the indicated days. Data shown represent means ± SD (error bars; control group, *n* = 10 or treatment groups, *n* = 6; *p < 0.05, **p < 0.01, ****p < 0.0001). **c** BT-474/R1-7 xenograft tumours were isolated, then signalling proteins were determined by western blotting. **d** Proposed model of T-DM1 resistance in BT-474/R1-7 cells.

Finally, to extend these in vitro findings to an in vivo setting, we performed xenograft studies. As shown in Fig. 6a, mono-treatment with either T-DM1 (3 mg/kg, *n* = 6) or dasatinib (3 mg/kg, *n* = 6) led to only modest inhibition of tumour growth (45.4 ± 15.7% and 27.6 ± 22.6%, respectively) in nude mice bearing BT-474/R1-7 xenografts, whereas combined treatment with both agents (*n* = 6) significantly improved tumour growth inhibition (89.2 ± 6.1%). In SK-OV-3/YES1 Y537F model, T-DM1 plus dasatinib led to a significant shrinkage of tumours (69.3 ± 1.7%, *n* = 6), whereas T-DM1 (3 mg/kg, *n* = 6) or dasatinib (3 mg/kg, *n* = 6) alone was not so effective (47.4 ± 5.5% and 30.1 ± 4.3%, respectively) (Fig. 6b). Moreover, no apparent toxicity was observed in either model (Fig. 6a, b). We further found that activation (phosphorylation) of the signalling proteins SFK, EGFR, Akt and Erk1/2 was significantly decreased and p27 was significantly increased by combinatorial treatment in BT-474/R1-7 xenografts, as shown in Fig. 6c and Supplementary Fig. 2e. Collectively, these results suggest that Yes inhibition by dasatinib sensitises BT-474/R1-7 and SK-OV-3/YES1 Y537F tumours to T-DM1 in vivo.

## Discussion

T-DM1 is a powerful HER2-targeted ADC that demonstrates remarkable efficacy in the clinic. However, after long-term treatment with this highly specific, targeted agent, tumour cells inevitably become reprogrammed so as to sustain cell survival.^25^ Here, we discovered that in a cell line incubated long term with T-DM1, Yes was overexpressed owing to amplification of chromosome region 18p11.32. Increased activated Yes subsequently activated multiple proliferation-related signalling pathways, and thereby conferred cross-resistance to HER2-targeted drugs (Fig. 6d). Notably, we demonstrated that inhibition of Yes with dasatinib reversed this resistance both in vitro and in vivo (Fig. 6d). These findings uncover a novel mechanism of T-DM1 resistance and provide a valuable reference for the development of rational strategies for overcoming resistance to HER2-targeted drugs in the clinic.

The tyrosine kinase Yes, encoded by the *YES1* gene, is a member of the SFK family. In addition to Yes, there are eight other highly homologous members of the SFK family, namely c-Src, Fyn, Lyn, Lck, Fgr, Blk, Hck and Yrk.^23^ SFKs activate multiple signalling proteins, including EGFR,^27^ PI3K/Akt, Ras/MAPK, FAK, RhoA, cortactin, p130CAS, paxillin, tensin-3, p27 and Bcl-2,^28^ and modulate a number of biological functions, such as cell proliferation, survival, differentiation, migration, invasion, adhesion, angiogenesis and immune cell function.^29^ Although the transforming ability of Yes has not been fully studied, some miRNAs were shown to regulate tumour progression via *YES1* regulation.^30,31^ Recently, a novel Yes inhibitor CH6953755 (more specific for Yes than other SFKs) was generated and showed potent antitumour activity against *YES1*-amplified cancers in vitro and in vivo.^32^ In this study, we found that phosphorylation of SFK, EGFR, Akt and Erk1/2 were significantly increased in *YES1*-amplified BT-474/R1-7 cells, and could be inhibited by dasatinib or siRNAs targeting Yes. Moreover, ectopic expression of Yes in different cancer cells led to resistance to HER2-targeted drugs. These results add evidences to *YES1* as an independent oncogene among SFK family members.

c-Src, the most studied SFK, has been implicated in conferring resistance to many targeted therapies, such as anti-EGFR therapies in lung cancer,^33^ antiandrogen therapies in prostate cancer,^34^ as well as anti-HER2 therapies (trastuzumab)^25^ and antioestrogen therapies^35^ in breast cancer. c-Src also modulates sensitivity to chemotherapeutic drugs, such as the antimicrotubule agent paclitaxel.^36,37^ Whereas c-Src has been extensively studied in the context of acquired drug resistance, comparatively less is known about Yes-induced drug resistance. It has been reported that *YES1* is amplified in clinical cases of acquired resistance to EGFR inhibitors^38^ and in osimertinib-induced resistant cell lines,^39^ but this conclusion was not directly confirmed by ectopically overexpressing Yes. We report here for the first time that Yes is overexpressed in breast cancer cells with acquired resistance to T-DM1 owing to amplification of chromosome region 18p11.32, and showed that this increase in Yes expression conferred resistance to different types of HER2-targeted drugs. In this study, we used comprehensive research strategies, including establishment of various cell lines stably transfected with wild-type or activated mutant version of *YES1*, to establish that activated Yes leads to resistance. This work provides novel insights into the role of Yes in the drug-resistance process, supported by solid evidence.

The *YES1* gene maps to chromosome region 18p11.32, which is reported to be frequently and extensively amplified in different cancers.^40–43^ In particular, *TYMS*, another gene that is closely linked to cancer, is located very near *YES1* in the 18p11.32 region. TYMS catalyses the methylation of dUMP to dTMP, and is a target for the chemotherapy drug 5-FU, one of the most commonly used drugs to treat cancer, including breast cancer.^44^ Notably, it has been found that *YES1* and *TYMS* are usually co-amplified and consequently co-overexpressed in cancer,^43,45^ findings similar to those of this study. Because patients treated with 5-FU are reported to exhibit a significantly greater frequency of *TYMS* amplification,^46^ *YES1* copy numbers should be determined in patients pretreated with 5-FU before initiating HER2-targeted therapy, given the current study’s finding that *YES1* amplification confers resistance to HER2-targeted drugs. Moreover, several regimens of HER2-targeted drugs (including T-DM1) in combination with 5-FU or capecitabine (5-FU pro-drug) have been studied in clinical trials.^47,48^ Specifically, trastuzumab + 5-FU/capecitabine + cisplatin has been approved as a first-line standard of care for HER2-positive metastatic gastric/gastroesophageal junction adenocarcinoma, and lapatinib + capecitabine has been approved for HER2-positive advanced or metastatic breast cancer with certain prior therapies, including an anthracycline, a taxane, or trastuzumab.^49^ Although these combinations exhibit enhanced antitumour activity, it is worth noting that under the dual selective pressure of HER2-targeted drugs and fluorouracil/capecitabine, there is a higher probability that amplification of chromosome region 18p11.32, where *YES1* and *TYMS* genes reside, will occur in these patients. Thus, monitoring copy numbers of these genes in the clinic is essential for obtaining additional statistical information and to avoid possible resistance in these patients. Interestingly, chemotherapies, such as adriamycin and taxol, displayed different sensitivity in *YES1* amplification (BT-474/R1-7) cells (Supplementary Fig. 4), and further investigation is warranted.

Dasatinib, which mainly targets SFKs and Abl, has been approved by the FDA for the treatment of chronic myeloid leukaemia and Philadelphia chromosome-positive acute lymphoblastic leukaemia. Despite being used clinically for many years, dasatinib has not yet been approved for the treatment of solid tumours; notable in this context, results from several clinical trials of dasatinib on different types of solid tumours have not been encouraging.^50–52^ However, it should be noted that most of these clinical trials were performed in unselected patients.^50–52^ In the current study, we demonstrated that cells with *YES1* amplification were remarkably more sensitive to dasatinib, which considerably reversed resistance to HER2-targeted drugs, even at low concentrations. Thus, *YES1* may potentially serve as a biomarker for the efficacy of dasatinib; moreover, dasatinib may be beneficial as monotherapy or mechanism-based combination therapy in solid tumours with *YES1* amplification. Further exploration of the therapeutic opportunities of dasatinib in solid tumours may prove promising.

In summary, we report a novel mechanism in which amplification of chromosome region 18p11.32, where the *YES1* gene resides, leads to overexpression of the SFK member Yes in cells incubated long term with T-DM1, and confers cross-resistance to HER2-targeted drugs. We further suggest that combined inhibition of HER2 and SFK could be a promising treatment strategy for overcoming resistance to HER2-targeted drugs. These findings provide novel insights into the treatment of resistance to HER2-targeted drugs, but still need clinical verification.

## Supplementary information

Supplementary Fig 1

Supplementary Fig 2

Supplementary Fig 3 and 4

Supplementary Material

## Data Availability

The data generated during the current study are available from the corresponding author upon reasonable request.
