# Supplementary figures and images for: YES1 amplification confers trastuzumab–emtansine (T-DM1) resistance in HER2-positive cancer
Source: Br J Cancer. 2020 Jun 23;123(6):1000–11. doi: 10.1038/s41416-020-0952-1 (PMC7494777; doi:10.1038/s41416-020-0952-1)

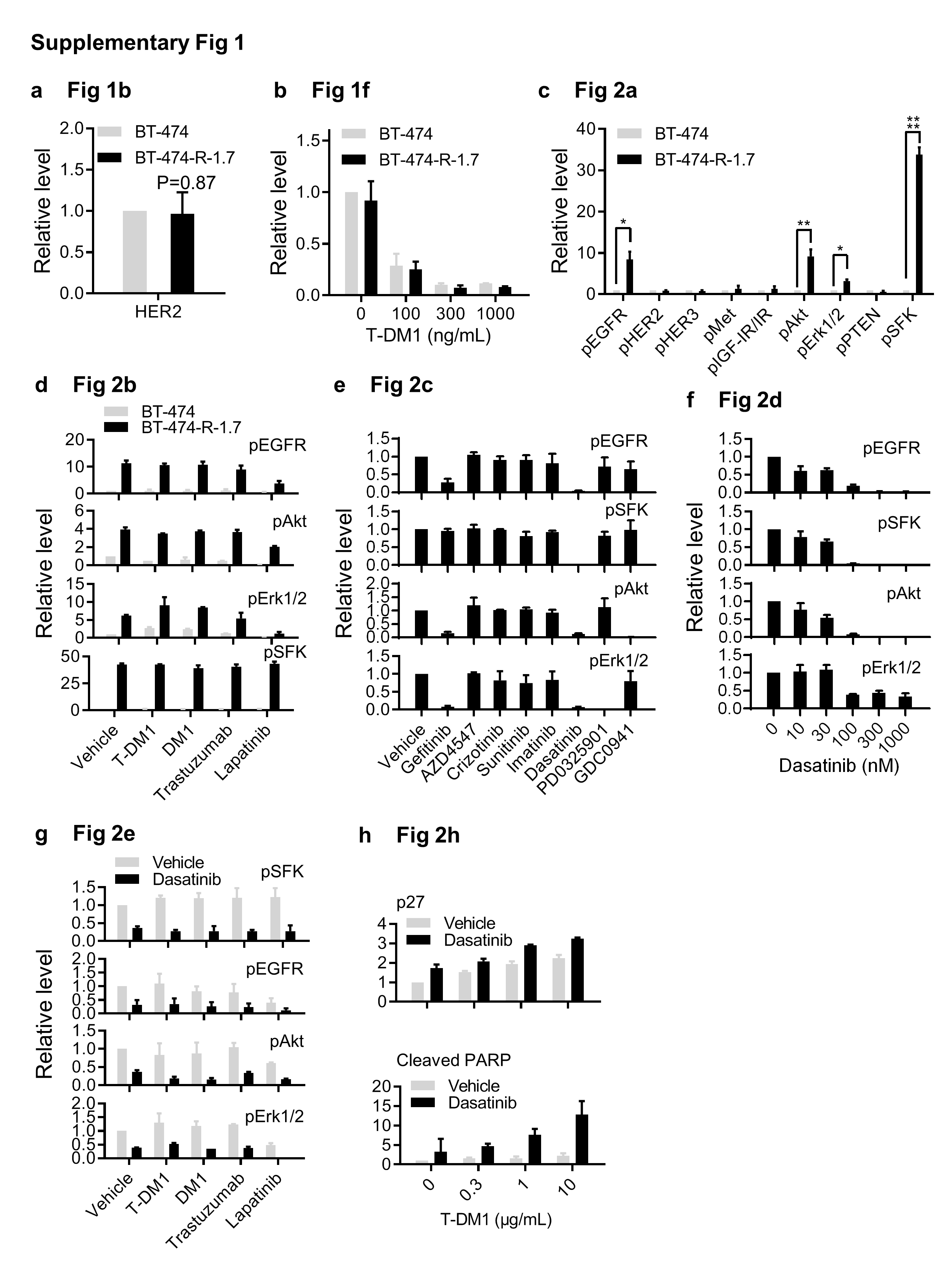

Supplement: Supplementary file 1 — Supplementary Fig 1 [file 41416_2020_952_MOESM1_ESM.tif]

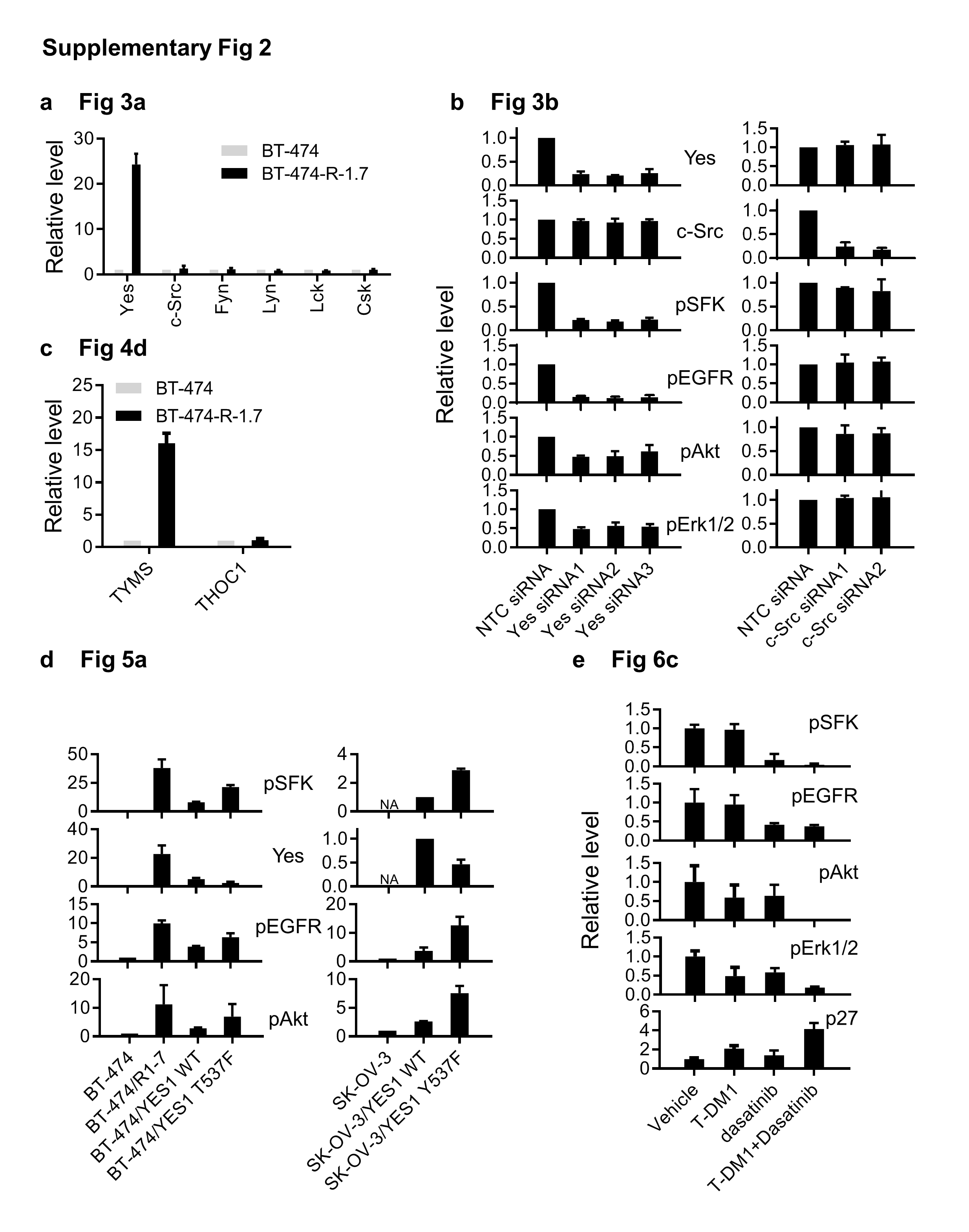

Supplement: Supplementary file 2 — Supplementary Fig 2 [file 41416_2020_952_MOESM2_ESM.tif]

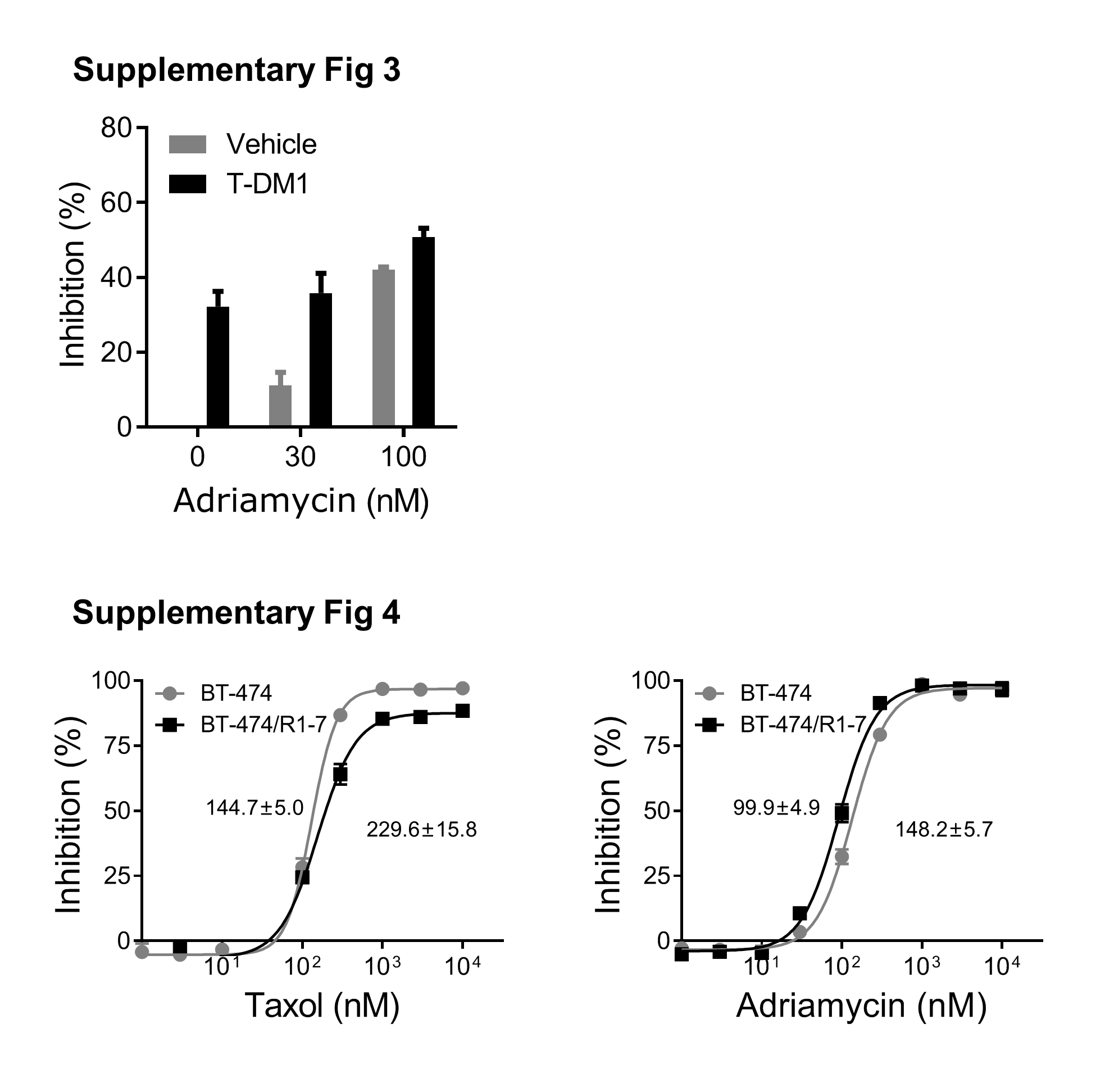

Supplement: Supplementary file 3 — Supplementary Fig 3 and 4 [file 41416_2020_952_MOESM3_ESM.tif]
