# Supplementary material for: YES1 amplification confers trastuzumab–emtansine (T-DM1) resistance in HER2-positive cancer
Source: Br J Cancer. 2020 Jun 23;123(6):1000–11. doi: 10.1038/s41416-020-0952-1 (PMC7494777; doi:10.1038/s41416-020-0952-1)
Supplement: Supplementary file 4 — Supplementary Material [file 41416_2020_952_MOESM4_ESM.docx]

***YES1* amplification confers trastuzumab-emtansine (T-DM1) resistance in HER2-positive cancer**

Lei Wang, Quanren Wang, Piaopiao Xu, Li Fu, Yun Li, Haoyu Fu, Haitian Quan*, Liguang Lou*

*Shanghai Institute of Materia Medica, Chinese Academy of Sciences, Shanghai 201203, China*

**Supplementary Materials and Methods**

**siRNA transfection.**

Cells were transfected with small interfering RNA (siRNA) duplexes using Lipofectamine 2000 (Thermo Fisher Scientific, Waltham, MA, USA) according to the manufacturer’s instructions. siRNAs with the following sequences were obtained from GenePharma (Shanghai, China):

**Supplementary Table 1.** siRNA sequences used in this study.

| Gene | siRNA | siRNA sequence（5'-3'） |
| --- | --- | --- |
| YES1 | siYES1#1 | CCCACUACAGUGUCACCAUTT |
|  | siYES1#2 | GGUGGAUACUAUAUCACAATT |
|  | siYES1#3 | GCACUGUAUGGUCGGUUUATT |
| SRC | siSRC#1 | GAAGCUGAGGCAUGAGAAGTT |
|  | siSRC#2 | CUUUGCACACCAGGUUCUCTT |
| CLUL1 | siCLUL1#1 | GCCACUCUUGGUGUUUAUUTT |
|  | siCLUL1#2 | CCCAAAUGGAGGAUGUGUUTT |
| TYMS | siTYMS#1 | ACAGAGAUAUGGAAUCAGATT |
|  | siTYMS#2 | CCUGACGACAGAAGAAUCATT |
| ENOSF1 | siENOSF1#1 | GCCAAAUCAUCCGAGACAUTT |
|  | siENOSF1#2 | GGACCAGGUUUAAAGUAAATT |

**RNA isolation and quantitative reverse transcription-PCR (qRT-PCR)**

RNA was isolated using the TRIzol reagent system and reverse-transcribed using the PrimeScript Reverse Transcription reagent Kit (Takara, Dalian, China). qPCR was performed on a StepOnePlus Real-Time PCR System (Thermo-Fisher Scientific, Sunnyvale, CA, USA) according to the instructions for the SYBR Premix Ex Taq II kit (Takara, Dalian, China). mRNA was quantified using the 2^-ΔΔCT^ method, and target mRNA levels were normalized to those of the housekeeping gene, *GAPDH*. The sequences of primers used for qRT-PCR analysis were as follows:

**Supplementary Table 2.** Primer sequences (RNA) for qRT-PCR used in this study.

| Gene | Forward sequence | Reverse sequence |
| --- | --- | --- |
| YES1 | GAGAATCTTTGCGACTAGAGG | CTGGCATCATTGTACCTGG |
| SRC | AGGCTGGCTTCTGCTGTTGA | CTGGGCCTTGGAATTTCGGA |
| FYN | CACCGTCTTTGGAGGTGTG | TCATCTTCTGTCCGTGCTTC |
| LYN | ATGTGAGAGATCCAACGTCC | TGCCATCATAGGGGTACAAG |
| BLK | CTTCAACCACCTTACTCCTCC | AGGTCCCGATCATTCATAGC |
| HCK | GGCCTAATAGCCACAACAGC | TGAGGTCTTCGTGGTGAATG |
| FGR | AACCCTGGCTTCCTTGATAG | TTGGTGAAGGTGAGGTCATC |
| THOC1 | AGCCAAGGAATTACCGCCTC | CTCGCCGAACATCAGGACTT |
| COLEC12 | CGGTTACAAGCGGTTTGGTAT | GCGAGATGTTTCCATGCCAC |
| CETN1 | TGATCTCCGAGGTGGACAGG | TTCCCGGTCTCATCGTCATC |
| CLUL1 | AGACAAAGCTCCTGACCACG | CTGTGTGCAGAGCAGGTACA |
| TYMS | GGGCAGATCCAACACATCC | GGTCAACTCCCTGTCCTGAA |
| ENOSF1 | TGGGCAGCTCAGATGGATTG | ACTTCCAGACAGGCTTTCCC |
| ADCYAP1 | GAAACCCGCTGCCAGACTTC | CGTGGGCGACATCTCTTCTC |
| GAPDH | GGGGAAGGTGAAGGTCGGAGTC | CAAGCTTCCCGTTCTCAGCCTT |

**DNA extraction and qPCR**

DNA was extracted using the DNeasy blood and tissue Kit (Qiagen, Hilden, Germany) according to the manufacturer’s instructions. qPCR was performed as described above. Target gene levels were normalized to those of the housekeeping gene, *RNaseP*. The sequences of primers used for qPCR analysis were as follows:

**Supplementary Table 3.** Primer sequences (DNA) for qPCR used in this study.

| Gene | Forward sequence | Reverse sequence |
| --- | --- | --- |
| USP14 | GATGGAGCGACCCTTCTTGG | AGGTTTACAATCTGCCTCGGG |
| THOC1 | AGTGGCGCAAAAGGTCTAGT | GAAGTTGGAGAGCCCTCAGT |
| COLEC12 | GCGATGCAATCGCTTTGACT | GACACCTAACGCCCTTGGAA |
| CETN1 | TTGATGACGATGAGACCGGG | TCCGAAGGAACTCCTCCTCG |
| CLUL1 | GCAGCAGCACAATTATCGGG | CCCACATTCCAGCATGTCCT |
| TYMS | GCCTCGGTGTGCCTTTCA | CGTGATGTGCGCAATCATG |
| ENOSF1 | AGGGACCCAAAAGCTTCGAG | AGACCCATCGGCAGTTTCTG |
| YES1 | TTACGGAATCATGCCACTC | CCCATGCCCAATAAAGTG |
| ADCYAP1 | ACGTTTTTCAACTCGGCGTG | GCGCTAAATTGTCCTCGCTG |
| METTL4 | CAGGAGAGGGGTGCACTAAC | GCACAATTGCTACCAGACCC |
| TGIF1 | ATGGCTGAGACAGAGGCAAC | CACCGAACCCTCAGCTTTCT |
| LAMA1 | TTGGGGGACAACCCTAGTCA | TGCAAATTCCGCCCACTACT |
| PTPRM | GACACAGGTGGATAGACGGC | GTTCCCAAACGCAGAGACCT |
| GACAT2 | AAACGTACTGCCACCTGAGC | TATGGGCACTGGCCAACATC |
| MTCL1 | AGCACTGGGTGCTTAATCCG | CTCTCAAGTCACCTGGATCGT |
| PPP4R1 | ACTGAGGGTAACCGCCTAGA | GCCACTACCAGATACAGCCC |
| RAB31 | TGAATTGTGCCAGCGTCTCT | CCGGACAATCGAGTTAGCGT |
| TUBB6 | TTTTCGATCTTGGCGAGTGC | AGAACCTTGTTACCCGGAGC |
| PTPN2 | AAAGGGAGTTGTGTCAGCGG | CAACGGAAAGCCGTTCAAGG |
| ROCK1 | TCCACTATTGTCCCTGTCGC | GGCAAGGCATACAGGGGATT |
| PIK3C3 | AGCACAATGGTATGCGGTGA | ATCCCTACCCCATCCGCTAA |
| MAPK4 | CCCTGGCTATATCACCCTCTTGTT | TATCAGTTTGGAGTGAGGGGCT |
| BCL-2 | CTGTTTTCCCCAGCCTGGAT | TCCCTCAAGAACAAGCGTCC |
| RNaseP | TGGGAAGGTCTGAGACTAGGG | CGTTCTCTGGGAACTCACCT |

**Supplemental figure legends**

**Supplemental Fig. 1.** BT-474/R1-7 cells were treated with different concentrations of adriamycin, with or without T-DM1 (300 ng/mL), for 120 h. Cell survival was measured using sulforhodamine B assays. Data shown represent means ± SD (error bars) from triplicates.

**Supplemental Fig. 2.** Cells were treated with different concentrations of taxol or adriamycin for 120 h, after which cell survival was measured using sulforhodamine B assays. IC_50_ was exhibited close to the corresponding dose-response curve. Data shown represent means ± SD of three independent experiments.

**Supplemental Fig. 3.** The quantitative graphs for Western blots in Fig. 1b, 1f, 2a, 2b, 2c, 2d, 2e and 2h were showed in a-h, respectively. *p < 0.05, **p < 0.01, ****p < 0.0001.

**Supplemental Fig. 4.** The quantitative graphs for Western blots in Fig. 3a, 3b, 4d, 5a and 6c were showed in a-e, respectively.
